# Health Behaviours and the Sense of Optimism in Nursing Students in Poland, Spain and Slovakia during the COVID-19 Pandemic

**DOI:** 10.3390/ijerph19042388

**Published:** 2022-02-18

**Authors:** Ewa Kupcewicz, Marzena Mikla, Helena Kadučáková, Daria Schneider-Matyka, Elżbieta Grochans

**Affiliations:** 1 Department of Nursing, Collegium Medicum University of Warmia and Mazury, 11-041 Olsztyn, Poland; ekupcewicz@wp.pl; 2 Department of Nursing, Campus de Ciencias, University of Murcia, El Palmar, 30120 Murcia, Spain; marzena.mikla@um.es; 3 Group ENFERAVANZA, Murcian Institute of Biosanitary Research (IMIB), 30120 Murcia, Spain; 4 Department of Nursing, Faculty of Health, Catholic University in Ruzomberok, 034-01 Ruzomberok, Slovakia; helena.kaducakova@ku.sk; 5 Department of Nursing, Pomeranian Medical University in Szczecin, 71-210 Szczecin, Poland; grochans@pum.edu.pl

**Keywords:** pandemic, COVID-19, dispositional optimism, health behaviours

## Abstract

(1) The COVID-19 outbreak brought about several socio-economic changes and has had a negative impact on the mental health of people around the world. The aim of this study was to examine the correlation between health behaviours and dispositional optimism among nursing students in Poland, Spain and Slovakia during the COVID-19 pandemic. (2) The study was carried out in spring 2021 on a group of 756 nursing students in Poland (*n* = 390), Spain (*n* = 196) and Slovakia (*n* = 170). The diagnostic survey method was applied with the questionnaire technique, and the following standardised research tools were used: Life Orientation Test—Revised (LOT-R) and the Health Behaviour Inventory (IZZ). The survey was conducted in a mixed arrangement: in direct contact with the respondents in Poland and in Spain and online in Slovakia. (3) Significant differences were demonstrated in health behaviour preferences in students in Poland, Spain and Slovakia (F = 6.22; *p* < 0.002). The highest IZZ index was found in Spanish students (82.60 ± 13.65), while lower values were observed in Slovak (80.38 ± 13.74) and Polish (78.44 ± 13.47) students. The correlation between dispositional optimism and a positive attitude was the strongest in the Polish sample (r = 0.56; *p* < 0.001), at a high level in the Spanish sample (r = 0.53; *p* < 0.001) and at an average level in the Slovak sample (r = 0.48; *p* < 0.001). (4) 1. The study showed a moderating effect of the country of origin on the students’ health behaviours. 2. Dispositional optimism is an important predictor of the students’ health behaviours, regardless of the country of origin.

## 1. Introduction

Health behaviour is a combination of knowledge, skills and attitudes, owing to which health-related activities can be initiated [1]. They are also defined as all health-related activities taken up by an individual, including pro-health and anti-health activities. Health behaviours generally denote health-related convictions, expectations, thoughts and motives. The scientific search for the factors with an impact on health behaviours has helped to develop numerous models for changes in health behaviours which emphasise personal beliefs about health, the sense of self-efficacy, motivation for change and the role of resources [2,3]. The function of the motivator for health behaviours is performed by health awareness [4], described as the knowledge and skills acquired through health education. Health behaviours can change throughout one’s life, but the juvenile period is of key importance [5]. Entering adulthood is associated with taking up and carrying out tasks in certain areas despite physical, social and psychological obstacles. Studying is a period in one’s life in which young people face difficult situations, which is why students are always regarded as a risk group with respect to taking up anti-health behaviours [6].

Cultural differences and people’s beliefs regarding compliance with recommendations during the pandemic are another factor with potentially a significant impact on health behaviours. Studies have shown that the level of social trust among Poles is low, which may be reflected in health behaviours during the COVID-19 pandemic [7]. Moreover, beliefs related to the coronavirus, which are contrary to medical knowledge and which are shared by a certain part of the population, are correlated negatively to the compliance with the recommendations on epidemic safety [8]. A study conducted in spring 2020 showed that Poland had one of the lowest levels of compliance with the regulations on applying behavioural measures (wearing face masks and keeping distance), whereas higher levels were observed in countries where the mortality rate was high, including Spain [9]. The level of Spaniards’ trust in healthcare professionals is high, and the media is the main source of information for them [10]. The personality traits were also found to have an impact on one’s behaviour and emotions. The study conducted during the two pandemic waves among respondents in Slovakia confirmed the existence of several important correlations between personality traits and behavioural and emotional reactions. It is also suggested that the second wave entailed a psychological adaptation to the pandemic [11]. Slovaks, with their high level of kindness, exhibiting an increased need for looking after others, complied with the health-related recommendations during the pandemic more frequently. Moreover, trust in the government can be a significant impulse to increase motivation for compliance [12].

Literature reports increasingly often stress the significant role of personal and social resources, whose type and level can affect one’s health behaviours. The many personal resources which are important for keeping oneself healthy include the level of dispositional optimism. A higher optimism level implies higher self-esteem, extraversion, a better sense of self-control and consolidation of one’s self-worth and self-efficacy. A higher optimism level also improves one’s life satisfaction as well as increases one’s desire and capabilities for facing adversities [13,14,15,16]. The concept of dispositional optimism was developed by Michael Scheier and Charles Carver (1992). In their view, dispositional optimism is a relatively constant human trait, independent of the temporal or situational context. It is a personality dimension which applies to one’s generalised expectations regarding the outcome of one’s actions. Dispositional optimism determines the choice of a goal and methods applied to achieve it. It stimulates motivation and perseverance in pursuing one’s goals, but it also facilitates decision-making in difficult situations [17,18]. It applies to the generalised expectation of positive events in one’s life, regardless of where one sees their causes. Positive events can result from personal actions, competence or other people’s favourable attitude. In this sense, it differs from such concepts as causal attributions or a sense of self-efficacy. The regulatory role of dispositional optimism manifests itself when one considers the possibility of attaining one’s goal, often in the context of obstacles which one may face on the way to attaining it. Dispositional optimism makes one believe that it will be attained [19]. Optimism is one of those resources which help one to achieve success in life because it makes one choose the means to pursue the goals more adequately [20,21]. When optimistic people form judgements regarding their own future, they perceive more possibilities of events associated with their success [22]. Dispositional optimism engages a subject on a cognitive level and applies to motivational processes—optimists make more efforts to pursue their personal goals and are more tenacious in it [23].

The COVID-19 outbreak brought about some socio-economic changes and has had a negative impact on the mental health of people around the world [24,25]. The restrictions imposed with respect to study, work, leisure, healthcare and transport have created new conditions for citizens of many countries. The unprecedented loss of social security with changes in everyday, professional and social life has created a need to examine the social consequences of the phenomenon in the context of students’ health behaviours, taking into consideration the ability to face adversities.

The aim of this study was to examine the correlation between health behaviours and dispositional optimism among nursing students in Poland, Spain and Slovakia during the COVID-19 pandemic.

The study aimed to answer the following research questions:

1. What health behaviours are exhibited, and what is the level of dispositional optimism among nursing students, considering inter-group differences?
2. To what extent is the level of dispositional optimism connected with health behaviours among nursing students, and what are the inter-group differences?
3. What role does dispositional optimism play, and which of the socio-demographic variables under study and those related to the lifestyle are more important in predicting health behaviours among nursing students?

## 2. Materials and Methods

### 2.1. Settings and Design

The study was conducted on a group of 756 nursing students in first degree (bachelor’s degree) studies between 20 March and 15 May 2021. The respondents had hybrid classes in the University of Warmia and Mazury in Olsztyn, Pomeranian Medical University in Szczecin (Poland), as well as in Murcia University (Spain) and in the Catholic University in Ružomberok (Slovakia). The students’ age under 30 years was the enrolment criterion, and students who refused to grant their informed and voluntary consent for participation in the study were excluded. After consent to the study was granted by the dean, the questionnaire sets were distributed by one researcher at each of the universities while maintaining the sanitary regime due to the COVID-19 pandemic. The survey was conducted in a mixed arrangement: during classes at the university in Poland and in Spain, whereas the questionnaire sets were sent to the students of the Slovak university by e-mail due to the sanitary restrictions. After being completed, they were returned by the same route to one of the researchers within two days. After being completed, they were returned by the same route to one of the researchers within two days. Students at each of the universities were informed about the aim and rationale behind the study and about the method of completing the questionnaires. They could also ask questions and receive detailed answers. They could withdraw from the study at any time without giving a reason. The study was anonymous and voluntary. It took approximately 10 min on average to complete the questionnaire. A total of 850 survey forms were distributed. After the material was collected and those completed incorrectly were eliminated, 756 (88.94%) were taken for the statistical analysis. The empirical data were collected in the database and encoded with Excel, and the results were analysed collectively.

This study meets the criteria of a cross-sectional study [26], and it is part of an international research project executed within one of the researcher’s (E.K.) scientific internship programmes. This project was given a favourable opinion (No. 3/2021) by the Senate Scientific Research Ethics Committee at the Olsztyn Higher School in Olsztyn and was carried out in accordance with the Declaration of Helsinki and the procedures and instructions in force at the universities.

### 2.2. Participants

The study included 756 nursing students from three European countries. There were 390 students (51.59%) in the study group in Poland, 196 (25.92%) in Spain and 170 (22.49%) in Slovakia. The mean participant age was 21.20 years (±1.97). Detailed characteristics of the participants are shown in Table 1.

### 2.3. Research Instruments

The diagnostic survey method was applied with the questionnaire technique, and the standardised research tools were used to collect the empirical data:

- Life Orientation Test—Revised (LOT-R) developed by Michael F. Scheier, Charles S. Carver, Michael W. Bridges [15];
- Health Behaviours Inventory—a Polish version developed by Zygfryd Juczyński [15].

An original survey questionnaire was used in the study to determine the socio-demographic variables, i.e., place of residence, gender, age, study year, as well as the subjective evaluation of the health status and selected elements of the lifestyle during the COVID-19 pandemic (i.e., physical exercise, meals, time spent working on a computer).

#### 2.3.1. Life Orientation Test—Revised (LOT-R)

The LOT-R scale contains 10 statements, with 6 of a diagnostic value for dispositional optimism and 4 buffer statements, with no bearing on the outcome. The respondents use a 5-point scale to evaluate how much a statement applies to them, from “it definitely does not apply to me”—0 points, to “it definitely applies to me”—4 points. The overall score is the sum of six statements, including three positive and three negative ones. The number of points for the negative statements was inverted before being summed up. The overall score ranged from 0 to 24 points. The higher the score, the higher the level of optimism. The raw score is converted to standardised units on the sten scale, which helps assess the intensity of dispositional optimism. Scores of 1–4 sten are regarded as low, indicative of proneness to pessimism. On the other hand, a score of 7–10 sten indicates a positive attitude. The Cronbach alpha for the original version is 0.78 [15].

#### 2.3.2. Health Behaviour Inventory (IZZ)

The IZZ scale contained 24 statements describing various health-related behaviours. The respondents identified the frequency of these health-related activities during the past year by assigning them points on a five-point scale, from “hardly ever”, to “nearly always”. The overall score was the sum of all the statements and it lay within an interval between 24 and 120 points. The higher the score, the greater the declared health behaviour intensity. After being converted into standardised units, the overall indices were interpreted according to the properties characterising the sten scale. Scores between 1 and 4 sten were regarded as low, whereas those from 7 to 10 sten as high, which corresponded to the area of ca. 33% of the lowest scores and the same percentage of the highest ones. Scores of 5 and 6 sten were regarded as average. The intensity of four categories of health behaviours were calculated separately, i.e., proper eating habits, prophylactic behaviours, a positive mental attitude and health-related practices. The intensity was indicated by the average number of points in each category, i.e., the score divided by 6. The IZZ internal consistency based on Cronbach alpha was 0.85 for the whole inventory, whereas it lay within an interval from 0.60 to 0.65 for its four subscales. The reliability evaluated by the test–retest method was 0.88 [15].

#### 2.3.3. Statistical Analysis

A statistical analysis of the data was performed with the Polish version of STATISTICA 13 (TIBCO, Palo Alto, CA, USA). The dataset analyses were performed to provide a description of individual attributes/variables, to examine relationships between variables and to verify statistical hypotheses. The socio-demographic variables were presented with the number of cases and percentage, and the group equipotency was verified with the chi-square test (χ^2^). The values of the parameters under study were shown with the mean, median, standard deviation, minimum and maximum. The intensity of dispositional optimism and health behaviours (low, average, high) among the students in the study group was shown on the sten scale, while the analyses of the result distribution differences in the subgroups were performed with the ANOVA (F). The Pearson correlation (r) was used to examine the power and direction of the correlation between dispositional optimism and health behaviours. A multiple regression analysis was performed in order to build a random variable estimation model from the independent variables. The interpretation of the correlation power between the analysed variables was based on the Guilford classification, taking, in sequence: |r| = 0—no correlation, 0.0 < |r| ≤ 0.1—slight correlation, 0.1 < |r| ≤ 0.3—weak correlation, 0.3 < |r| ≤ 0.5—average correlation, 0.5 < |r| ≤ 0.7—high correlation, 0.7 < |r| ≤ 0.9—very high correlation, 0.9 < |r| < 1.0—nearly full correlation, |r| = 1—full correlation. The test probability at the level of significance of *p* < 0.05 was taken as significant [15,27].

## 3. Results

### 3.1. Health Behaviours and Dispositional Optimism Intensity in Polish, Spanish and Slovak Nursing Students

Considering the frequency of individual health behaviours included in the IZZ, the overall intensity of such behaviours among nursing students during the previous year was established. The statistical analysis demonstrated significant differences in health behaviour preferences in students in Poland, Spain and Slovakia (F = 6.22; *p* < 0.002). The value of an overall health behaviour intensity index was the highest for the Spanish sample: 82.60 (±13.65) on a scale from 24 to 120 points. These values were lower in the Slovak (80.38 ± 13.74) and the Polish (78.44 ± 13.47) samples (Table 2).

**Table 2:** Health behaviours vs. intensity of dispositional optimism among the respondents in the study group—results of different significance tests, taking into account the grouping variable country of residence.

Variables	Country of Residence			ANOVA (F)	*p*-Value
	Poland—A *n* = 390 (51.59%)	Spain—B *n* = 196 (25.93%)	Slovakia—C *n* = 170 (22.49%)		
	M ± SD, Me, Min.–Max.	M ± SD, Me, Min.–Max.	M ± SD, Me, Min.–Max.		
Overall health behaviour	78.44 ± 13.47, 78 43–120,	82.60 ± 13.65, 83 46–110	80.38 ± 13.74, 81 36–118	6.22	0.002 *A* < *B,C* ***
Proper eating habits	3.23 ± 0.81, 3.17 1–5	3.29 ± 0.92, 3.50 1–5	3.19 ± 0.74, 3.17 1–5	0.73	0.47
Prophylactic behaviours	3.41 ± 0.71, 3.50 2–5	3.66 ± 0.72, 3.67 2–5	3.42 ± 0.75, 3.50 1–5	8.04	0.0003 *A,C* < *B* ***
Positive mental attitude	3.32 ± 0.74, 3.33 1–5	3.56 ± 0.71, 3.50 2–5	3.59 ± 0.69, 3.67 1–5	11.56	0.0001 *A* < *B* *** *A* < *C* ***
Health practices	3.11 ± 0.71, 3.17 1–5	3.26 ± 0.64, 3.33 1–5	3.20 ± 0.77, 3.17 1–5	2.97	0.051
Life Orientation Test—Revised	14.02 ± 4.48, 14 0–24	14.18 ± 4.30, 14 0–22	15.60 ± 3.87, 16 3–24	8.36	0.0003 *A,B* < *C* ***

**Figure 1:**
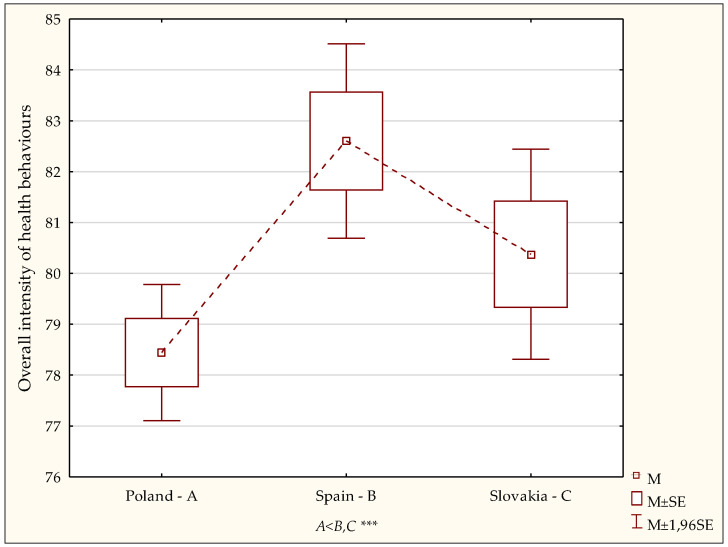
Overall intensity of health behaviours–diversity of scores. Statistically significant: *** *p* < 0.001.

Statistically significant: *** *p* < 0.001. The detailed analyses with the post hoc (LSD) test confirmed that the overall intensity of health behaviours in students in Poland was significantly lower than in students in Spain (*p* < 0.0001) or Slovakia (*p* < 0.0001) (Figure 1).

The overall health behaviour index was converted to standardised units on the sten scale and significant differences were shown to exist between the subgroups under comparison regarding the distribution of low, average and high scores (χ^2^ =219.49; *p* < 0.0002). The scores for these three subgroups are listed in Table 3. There were 44.62% of low scores in the Polish sample, 40.00% in the Slovak sample and 30.61% in the Spanish sample. There were only 15.90% of high scores (indicative of the high intensity of declared health behaviours) in the Polish sample, 22.94% in the Slovak sample and 28.06% in the Spanish sample.

**Figure 2:**
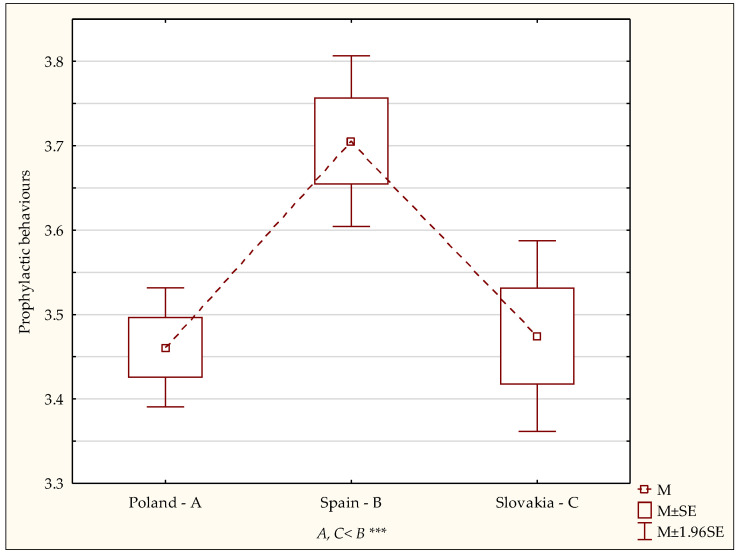
Intensity of prophylactic behaviours—diversity of scores. Statistically significant: *** *p* < 0.001.

Further statistical analyses estimated the intensity of health behaviours in four other categories, i.e., proper eating habits, prophylactic behaviours, health practices and positive mental attitude. Statistically significant differences between the samples under comparison, taking into the grouping variable (country of residence), were present in only two categories of health behaviours and they concerned prophylactic behaviours (F = 8.04; *p* < 0.0003) and positive mental attitude (F = 11.56; *p* < 0.0001; Table 2). Detailed analyses with the post hoc (LSD) test showed that the prophylactic behaviour index associated with observing health-related instructions and obtaining information on health and illness was significantly lower in the Polish (*p* < 0.0001) and Slovak (*p* < 0.0001) students compared to the Spanish students (Figure 2).

**Figure 3:**
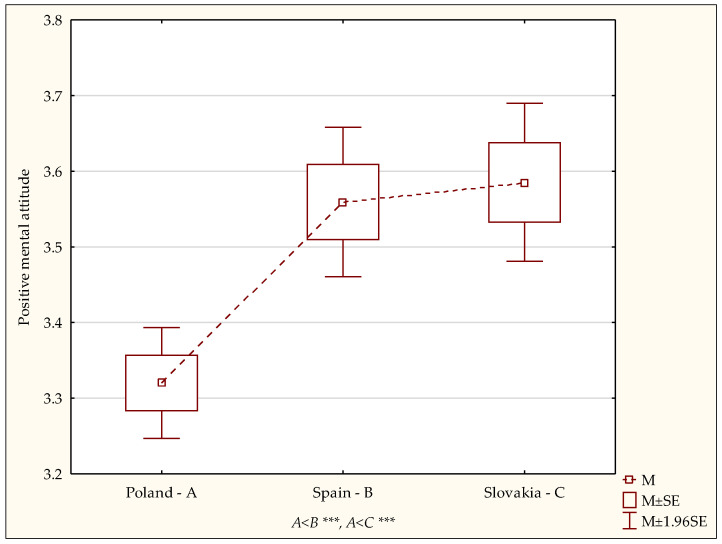
Intensity of positive mental attitude–diversity of scores. Statistically significant: *** *p* < 0.001.

Regarding the health behaviour category referred to as positive mental attitude, characterised by avoiding too strong emotions, stress and tension, it was demonstrated with a post hoc (LSD) test that the positive mental attitude index was significantly lower in the Polish sample than in the Spanish (*p* < 0.0001) or Slovak (*p* < 0.0001) samples (Figure 3).

**Figure 4:**
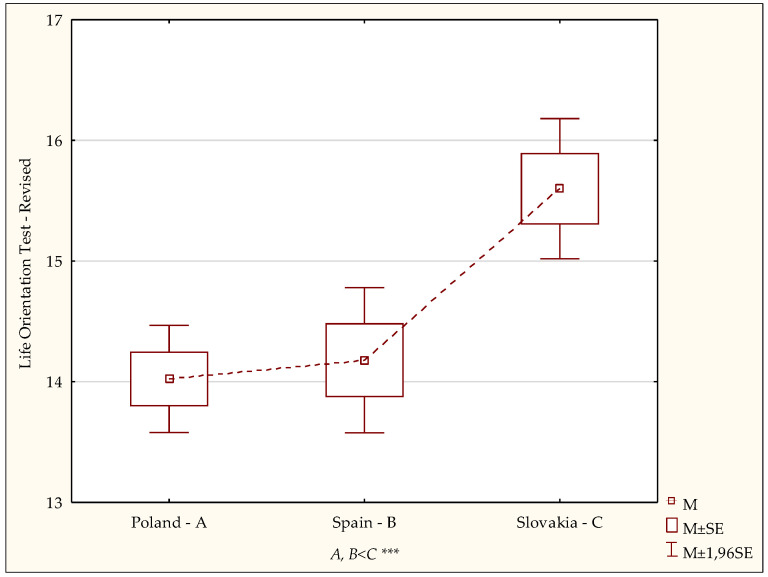
Intensity of dispositional optimism—diversity of scores. Statistically significant: *** *p* < 0.001.

The data in Table 2 show that the scores for the samples under analysis are diverse (F = 8.36; *p* < 0.0003). The mean level of optimism was the highest in the group of nursing students in Slovakia (15.60 ± 3.87). Detailed analysis with the post hoc (NIR) test showed that the dispositional optimism indexes for students in Poland (*p* < 0.0001) and Spain (*p* < 0.0001) were significantly lower than the index for students in Slovakia (*p* < 0.0001) (Figure 4).

The analysis of dispositional optimism confirmed the presence of statistically significant differences between the subgroups under comparison in the distribution of low, average and high scores (χ^2^ =19.61; *p* < 0.0006).

The highest percentage of low scores, indicative of proneness to pessimism, was noted among the Polish students (38.38%). The structure of scores shows that the percentage of people with a highly optimistic attitude was the highest in the Slovak sample (42.94%) and significantly lower in the Polish sample (32.05%) and the Spanish sample (29.08%) (Table 3).

### 3.2. Correlation between the Intensity of Dispositional Optimism and Health Behaviours in Polish, Spanish and Slovak Nursing Students

**Figure 5:**
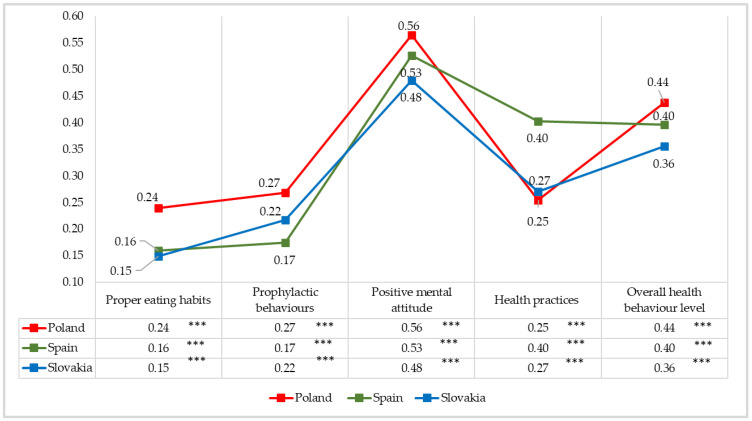
The nature and power of correlation between the intensity of dispositional optimism and health behaviours in the study participants—Pearson correlation coefficients (r). Statistically significant; *** *p* < 0.001.

The statistical analysis of the scores showed a significant correlation between the intensity of dispositional optimism and overall health behaviours and revealed the link between the intensity of dispositional optimism and health behaviours in the four categories under analysis, i.e., proper eating habits, prophylactic behaviours, positive mental attitude and health practices. These are significant positive correlations, which means that the higher the dispositional optimism level, the higher the intensity of overall health behaviours and the behaviours in individual categories. The correlation was the strongest between dispositional optimism and health behaviours in the positive mental attitude category, with high scores in the Polish sample (r = 0.56; *p* < 0.001) and in the Spanish sample (r = 0.53; *p* < 0.001) and average scores in the Slovak sample (r = 0.48; *p* < 0.001). These are positive correlations, which means that the more frequently the students exhibit optimistic attitudes, the better they cope in everyday lives, including such mental factors in the behaviours as avoiding too strong emotions, stress and tensions, or situations with a depressing impact. The lowest correlation coefficients (r = 0.24 to r = 0.15) were noted between dispositional optimism and health behaviours in the proper eating habits category, mainly taking into account the type of food (e.g., wholemeal bread, fruit and vegetables). The other results describing the power of correlation between the intensity of dispositional optimism and health behaviours in students in the Polish, Spanish and Slovak study are listed in Figure 5.

### 3.3. Predictors of Overall Health Behaviours among Polish, Spanish and Slovak Nursing Students

Health behaviours determined by the IZZ were noted when building the multiple regression model with a dependent variable, whereas the pool of independent variables included dispositional optimism, a subjective health status assessment, socio-demographic variables (gender, year of studies) and selected lifestyle elements (time spent working on a computer, number of meals and their regularity, the extent to which one’s physical exercise is restricted during the COVID-19 pandemic). The regression analysis for the Polish sample showed five variables to be predictors of health behaviours, which explained 34% of the score variation altogether. The share of dispositional optimism was the largest, and it explained 19% of the score variation. The second variable regularity of meals explained 7% of the score variation. The other three variables (subjective health status assessment, number of meals and year of studies) had an 8% share in the prediction of health behaviours in the group of Polish students.

Four variables were predictors of health behaviours among the nursing students in Spain, and they explained 34% of the score variation. The variable subjective health status assessment had the greatest share in health behaviour prediction as it explained 16% of the score variation. Another two variables (dispositional optimism and meal regularity) explained 7% of the score variation each. Restriction of physical exercise during the COVID-19 pandemic had a 4% share in the prediction of health behaviours among the Spanish students.

The regression analysis identified three predictors of health behaviours in the Slovak sample, which together explained 20% of the score variation. The first position was occupied by dispositional optimism, which explained 13% of the score variation, followed by restriction of physical exercise during the pandemic 4%, and the number of meals 3%. (Table 4).

## 4. Discussion

The COVID-19 pandemic poses a major threat to public health around the world and has had a considerable impact on mental health and well-being [28,29]. Restrictions regarding staying at home have disrupted everyday activities and affected certain groups of the population, such as university students, who were forced to adapt to online classes, thereby reducing interpersonal interactions and having a negative impact on their health behaviours.

An analysis of the results of the current study showed significant differences regarding the preferred health behaviours among Polish, Spanish and Slovak students. The highest health behaviour index was noted for the students in Spain compared to those in Poland and Slovakia. The smallest group of Spanish students obtained the overall health behaviour index at a low level compared to the students in the other two countries. The smallest group with a high level were students in Poland, and the highest health behaviour index levels were found in Spanish students. Additionally, the current study found significant differences with respect to the students’ country of origin in the categories of prophylactic behaviours and a positive mental attitude. The prophylactic behaviour index associated with observing health-related instructions and obtaining information on health and illness was significantly lower in Polish and Slovak students compared to Spanish students. When it comes to the health behaviour category referred to as positive mental attitude, characterised by avoiding overly strong emotions, stress and tensions, significantly lower scores were noted among the Polish students than in the Spanish and Slovak group.

It is worth noting that the study disregarded the pandemic situation, the social restrictions and the economic consequences in individual countries during the study [30,31,32], which can determine the health behaviours and the mental condition of the study participants. Moreover, studies conducted by Makowska et al. among adult Poles demonstrated that trust in the healthcare system and pharmaceutical companies could have an impact on health behaviours. Individuals exhibiting a higher level of trust were shown to comply, to a greater extent, with pro-health recommendations during the COVID-19 pandemic [33]. It was shown in another study that 36.7% of Poles placed great trust in medicine during the second wave of the pandemic, 36.3% of them reported moderate trust, and 27.1% reported low trust, which is a considerable group. This may be a consequence of the low level of Poles’ trust in medicine even before the health crisis [34,35,36]. 

The study published by YouGOV shows that Poles exhibit the lowest level of trust in the healthcare system among all the countries under study. Among the four variables: government, media, friends and family, and healthcare personnel, the latter enjoyed the greatest trust among the Spanish population, which can be explained by a higher health behaviour rate among the Spanish students [37]. Moreover, it was found on the basis of a literature review that cultural capital was significantly correlated with some health and lifestyle-related behaviours [38]. Women living in small towns with a worse financial standing, who were religious and less satisfied with their health status and who had no experience of a coronavirus infection in their closest circle showed low trust in the healthcare system [33]. The Spanish study confirmed a correlation between mental stress and a poor assessment of one’s health status. Variables were identified which predisposed the study participants to mental stress: being a female, young age, difficult professional situation, perceiving one’s health status as bad, close contact with an infected individual, as well as contact with individuals suspected to be so, seeking information about the pandemic situation frequently, loneliness and low sleep quality [39,40].

A study by Grande and Doye Baker showed that Canadian students demonstrated a moderate level of fear associated with the COVID-19 pandemic, and those with a high level of fear reported more negative choices concerning health behaviours. These findings emphasised that continuous restrictions during the COVID-19 pandemic have a negative impact on students’ health behaviours [41]. Similar observations were made in the study by Savage et al. that the COVID-19 pandemic has a negative impact on the mental health and physical exercise of British university students [42]. The findings of other studies also show a high frequency of mental health disorders during the pandemic [43,44,45,46].

A study conducted by Kropornicka et al. among medical students showed that nearly half of the respondents presented average health behaviours, with those coming from rural areas exhibiting more beneficial behaviours of this kind [47].

An analysis of the results of the current study concerning an assessment of the overall intensity of dispositional optimism among the nursing students during the COVID-19 pandemic showed the level of dispositional optimism in students in Poland and Spain to be significantly lower than in those in Slovakia. The highest percentage of low scores, indicative of proneness to pessimism, was noted among the Polish students. The highest percentage of highly optimistic students was found in the Slovak group, followed by the Polish and Spanish groups.

A higher optimism level implies higher self-esteem, extraversion, a better sense of self-control and consolidation of one’s self-worth and self-efficacy. It also improves one’s life satisfaction, and it increases one’s desire and capability for standing up to adversities [15,16,17]. The findings of a study by Jaworski et al. demonstrate that personality-related variables, such as positive thinking, coexisting with some variables related to a place of work, can reduce the risk of rationing nursing care, thereby affecting the care quality [48]. A study conducted by Sławska among Polish nursing students demonstrated a high level of dispositional optimism in over 40% of them, which was associated with better marks and improved studying results [49]. Dziąbek et al. [50] demonstrated high levels of dispositional optimism in approx. 36% of the nurses participating in the study moderate scores in approx. 39% and low in 24.07% [49].The findings of a study by Sprynska et al. showed some differences regarding dispositional optimism among Polish and Ukrainian students: Ukrainian students were more optimistic than Polish students [51]. According to the findings of the study conducted by Gustems-Carnicer et al., Spanish students showed that dispositional optimism is positively correlated with mental well-being and negatively correlated with mental distress [52]. In another study, conducted by Barhaghtalab et al., examining the correlation between optimism and health, it was found that highly optimistic individuals were often active, creative and hardworking, while at the same time being good initiators and planners. Moreover, such individuals can make the best of a stressful situation, which makes them less susceptible to further risks and pressure of stressful events. The immunity system is more effective in optimistic people, and they are more capable of coping with stress by applying more effective coping strategies for difficult situations [53].

To summarise, one can conclude that students with a higher level of dispositional optimism also declared a higher intensity of health behaviours, both in general and in individual categories. The strongest correlation was observed between dispositional optimism and a positive mental attitude, which was high in the group of students in Poland and average in the Slovak group. The more frequently the students exhibit optimistic behaviours, the better they cope in everyday lives, including such psychological factors in the behaviours as avoiding too strong emotions, stress and tensions, or situations with a depressing impact. The lowest correlation coefficients were noted between dispositional optimism and health behaviours in the proper eating habits category, mainly considering the type of food.

The study conducted by Bodys-Cupak et al. showed a high and average level of optimism in a majority of the nursing students participating in the research. Those with a higher level of dispositional optimism coped with stress significantly more often by active coping, positive redefinition, planning, acceptance and seeking emotional and instrumental support [54]. A study by Joseph et al. [55] showed similar findings. A low level of optimism in nursing students is significantly correlated with more frequent coping by denial, withdrawing from action, blaming oneself or using psychoactive substances. Soares et al. also demonstrated a correlation between a low optimism level and using psychoactive substances [56].

The analysis of the study findings attempted to identify the predictors of overall health behaviours among nursing students. One can conclude that the main role in the prediction of the health behaviours among nursing students in Poland and Slovakia is played by dispositional optimism, whereas a subjective health status assessment is the main determinant of health behaviours among students in Spain, followed by dispositional optimism.

### Study Limitations and Implications for Professional Practice

The authors note some limitations. The sample was not representative of the nursing students in the countries where the study was conducted. Additionally, the technique may have had an impact on the study findings. Firstly, it was conducted in a mixed arrangement: stationary in Poland and Spain, and online in Slovakia. Using an online questionnaire may result in sample bias towards more digitally competent respondents, who may also be less motivated. Secondly, no cultural adaptation of the health behaviour questionnaire in Spain or Slovakia was performed.

The study results can be useful in planning and adjusting interventions aimed at changing nursing students’ health behaviours from bionegative to biopositive. Dispositional optimism can play a considerable role in such health behaviour change. There are reasons for developing dispositional optimism in students, which also increases readiness for initiating health-promoting activities.

## 5. Conclusions

Positive correlations of various power between health behaviours and dispositional optimism were demonstrated among nursing students in Poland, Spain and Slovakia.

A moderating effect of the country of origin on nursing students’ health behaviours was demonstrated.

Dispositional optimism was shown to be an important predictor of the students’ health behaviours, regardless of the country of origin.

There is a need for reinforcing an optimistic outlook on life and the adoption of health-promoting activities by nursing students during the COVID-19 pandemic.

## Figures and Tables

**Table 1:** Characteristics of the participants.

Variables		Country of Residence N = 756		
		Poland—A *n* = 390	Spain—B *n* = 196	Slovakia—C *n* = 170
		*n* (%)	*n* (%)	*n* (%)
Gender	female	357 (91.54)	161 (82.14)	164 (96.47)
	male	33 (8.46)	35 (17.86)	6 (3.53)
Study year	first	140 (35.90)	28 (14.29)	55 (32.35)
	second	160 (41.03)	73 (37.24)	61 (35.88)
	third	90 (23.08)	95 (48.47)	64 (37.65)
Age (years)	≤20	128 (51.28)	63 (32.14)	71 (41.76)
	21–22	200 (44.18)	29 (14.80)	36 (20.59)
	≥23	62 (15.90)	126 (16.67)	126 (16.67)
Place and form of residence	with family/someone close	297 (76.15)	192 (97.96)	141 (82.94)
	on their own	93 (23.85)	4 (2.04)	29 (17.06)
Time of work on a computer (hours)	≤5	174 (44.62)	51 (26.02)	102 (60.00)
	6–9	135 (34.62)	84 (42.86)	55 (32.35)
	≥10	81 (20.77)	61 (31.12)	13 (7.65)
Number of meals	1.2	32 (8.21)	5 (2.55)	10 (5.88)
	3	174 (44.62)	47 (23.98)	45 (26.47)
	4	125 (15.13)	94 (47.96)	74 (43.53)
	≥5	59 (19.84)	50 (25.51)	41 (24.12)
Restriction of physical exercise during the pandemic	no	89 (22.82)	69 (35.20)	43 (25.29)
	yes, to a small extent	66 (16.92)	51 (26.02)	54 (31.76)
	yes, to a moderate extent	129 (33.08)	55 (28.06)	50 (29.41)
	yes, to a considerable extent	106 (27.18)	21 (10.71)	23 (13.53)
Subjective health status assessment during the pandemic	Bad	9 (2.31)	4 (2.04)	2 (1.28)
	good/average	257 (65.90)	118 (60.20)	114 (67.06)
	very good	124 (31.79)	74 (37.76)	54 (31.76)
Restriction of social contacts during pandemic	very high	82 (21.03)	98 (50.00)	36 (21.18)
	considerable	155 (39.74)	39 (19.90)	53 (31.18)
	moderate/average	83 (21.28)	56 (28.57)	49 (28.82)
	to a small extent	70 (17.95)	3 (1.53)	32 (18.82)

Explanation: N—number of group members; *n*—number of subgroup members.

**Table 3:** Diversity of scores for overall health behaviours and dispositional optimism on the sten scale in the Polish, Spanish and Slovak studies.

Variables	Results on A Sten Scale (1–10)	Country of Residence			Chi-Square Test χ2	*p*-Value
		Poland—A *n* = 390 (51.59%)	Spain—B *n* = 196 (25.93%)	Slovakia—C *n* = 170 (22.49%)		
		%				
Health Behaviour Inventory	Low (1–4)	44.62	30.61	40.00	219.49	0.0002 ***
	Average (5–6)	39.49	41.33	37.06		
	High (7–10)	15.90	28.06	22.94		
Life Orientation Test–Revised	Low (1–4)	38.38	31.12	18.82	19.61	0.0006 ***
	Average (5–6)	32.56	39.80	38.24		
	High (7–10)	32.05	29.08	42.94		

Statistically significant: *** *p* < 0.001.

**Table 4:** Predictors of overall health behaviours among Polish, Spanish and Slovak nursing students: Multiple regression results.

Variables		R^2^	*ß*	*p*-Value
Poland	Constant value			0.0001 ***
	Dispositional optimism	0.19	0.32	0.0001 ***
	Regularity of meals	0.26	0.20	0.0001 ***
	Subjective health status assessment	0.31	0.23	0.0001 ***
	Number of meals	0.33	0.14	0.001 ***
	Study year	0.34	0.12	0.01 *
	R = 0.59; R^2^ = 0.35; corrected R^2^ = 0.34			
Spain	Constant value			0.0001 ***
	Subjective health status assessment	0.16	0.21	0.001 ***
	Dispositional optimism	0.23	0.28	0.0001 ***
	Regularity of meals	0.30	0.23	0.0002 ***
	Restriction of physical exercise during the pandemic	0.34	−0.22	0.001 ***
	R = 0.60; R^2^ = 0.35; corrected R^2^ = 0.34			
Slovakia	Constant value			0.0001 ***
	Dispositional optimism	0.13	0.26	0.0001 ***
	Restriction of physical exercise during the pandemic	0.17	−0.18	0.02 *
	Number of meals	0.20	0.17	0.02 *
	R = 0.48; R^2^ = 0.23; corrected R^2^ = 0.20			

Statistically significant: * *p* < 0.05: *** *p* < 0.001.

## Data Availability

The data presented in this study are available on request from the first author.
